# Clinician-Reported Person-Centered Culturally Responsive Practices for Youth with OCD and Anxiety

**DOI:** 10.3390/children12081034

**Published:** 2025-08-07

**Authors:** Sasha N. Flowers, Amanda L. Sanchez, Asiya Siddiqui, Michal Weiss, Emily M. Becker-Haimes

**Affiliations:** 1 Department of Psychology, George Mason University, Fairfax, VA 22030, USA; asanch49@gmu.edu (A.L.S.); asiddi9@gmu.edu (A.S.); 2 Department of Public Health Policy and Management, School of Global Public Health, New York University, New York, NY 10003, USA; mw5652@nyu.edu; 3 Department of Psychiatry, University of Pennsylvania, Philadelphia, PA 19104, USA; emily.haimes@pennmedicine.upenn.edu; 4 Hall Mercer Community Mental Health, University of Pennsylvania Health System, Philadelphia, PA 19104, USA

**Keywords:** adaptation, augmentation, cultural responsiveness, pediatric anxiety, pediatric OCD

## Abstract

**Highlights:**

**What are the main findings?**

- Clinicians working in community mental health clinics described the need to understand their clients’ culture and context and noted ways in which they addressed these factors.
- Clinicians described incorporating culture and context through adapting existing treatment techniques, augmenting treatment with strategies not traditionally included in Ex-CBT, and utilizing process-based approaches.

**What is the implication of the main finding?**

- Learning from practicing clinicians’ approaches and challenges can help to inform clinical recommendations for more person-centered culturally responsive anxiety and OCD treatment for minoritized youth.

**Abstract:**

**Background:** Exposure-based cognitive behavioral therapy (Ex-CBT) is widely seen as the gold-standard treatment for anxiety and obsessive-compulsive disorder (OCD). Yet, minoritized youth are underrepresented in efficacy studies, raising questions about the applicability of Ex-CBT to minoritized youth. Effectiveness data suggest systematic adaptation of Ex-CBT to address youth culture and context is likely needed, and many clinicians make adaptations and augmentations in practice. However, research on the specific strategies clinicians use to address their youth clients’ culture and context within anxiety and OCD treatment is lacking. In the current study, we assess practice-based adaptations, augmentations, and process-based approaches utilized when delivering treatment to youth for OCD and anxiety in public mental health clinics. **Methods:** We conducted qualitative interviews with 16 clinicians from both specialty anxiety and general mental health clinics serving youth with anxiety or OCD in the public mental health system. Participating clinicians had a mean age of 32.19 (SD = 5.87) and 69% of therapists identified as female; 69% identified as White, 25% identified as Asian, and 6% as Black or African American. In qualitative interviews, clinicians shared how they addressed clients’ culture and context (e.g., social identities, stressors and strengths related to social identities and lived environment). Thematic analysis identified the strategies clinicians employed to address culture and context. **Results:** Clinicians reported incorporating culture and context through process-based approaches (e.g., building trust gradually, considering clients’ social identity stressors, engaging in self-awareness to facilitate cultural responsiveness) and through culturally adapting and augmenting treatment to promote person-centered care. Core strategies included proactive and ongoing assessment of clients’ cultural and contextual factors, adapting exposures and augmenting Ex-CBT with strategies such as case management and discussion of cultural context, and taking a systems-informed approach to care. **Conclusions:** Examining practice-based adaptations, augmentations, and process-based approaches to treatment for minoritized youth with OCD or anxiety can inform efforts to understand what comprises person-centered culturally responsive Ex-CBT. Empirical testing of identified strategies is a needed area of future research.

## 1. Introduction

Exposure-based cognitive behavioral therapy (Ex-CBT) is the current gold-standard treatment for anxiety, obsessive-compulsive disorder (OCD), and related disorders in youth [1,2,3]. Ex-CBT refers to treatment in which clients are gradually and systematically exposed to feared situations to help them reduce anxiety and compulsive behaviors. However, Ex-CBT suffers a significant research-to-practice gap such that up to 90% of clinicians treating anxious youth do not consistently utilize exposures in practice [4,5,6]. Furthermore, Ex-CBT is rarely offered in community-based settings where most minoritized youth (i.e., youth who face marginalization due to their social identities) receive care [6,7,8,9].

Additionally, the applicability of Ex-CBT to minoritized youth is in question, as these youth are underrepresented in efficacy trials [1,10]. Moreover, some studies demonstrate diminished treatment effects for racially, ethnically, and socioeconomically minoritized youth [11,12,13]. One potential explanation for these diminished effects is that treatment techniques addressing culture (e.g., salient social identities, beliefs about mental health and treatment, values) and context (e.g., stressors and strengths related to social identities and lived environment) have not been systematically integrated into Ex-CBT protocols. A recent review of evidence-based treatment (EBT) protocols for youth internalizing disorders found that less than 2% of modification guidelines address patient cultural factors [14]. Importantly, emerging work suggests Ex-CBT can be just as effective for racially and ethnically minoritized youth as it is for White youth when Ex-CBT is supplemented with cultural adaptations and augmentations [15,16,17].

Adaptation refers to the intentional modification of interventions to enhance their relevance, accessibility, and effectiveness for a particular client or population [18,19,20,21]. Cultural adaptation specifically emphasizes integrating culture into EBTs; for example, this may include translating materials into the client’s primary language, incorporating culturally relevant metaphors, symbols, or narratives, and ensuring that therapy goals reflect culturally specific values and belief systems [22]. Culturally adapted protocols tend to focus on a particular aspect of identity: namely, racial/ethnic identity. For example, researchers adapted trauma treatment for Hispanic or Latine/x youth by including stories with characters of the same ethnicity as the participants to foster their ability to identify with the characters [23]. A specific type of adaptation called augmentation refers to incorporating additional tools and supports that are typically not included among standard treatment components (rather than just adjusting existing treatment components) to address clients’ needs. This can involve incorporating discussion of topics particularly salient to a specific cultural group (e.g., addressing stressors related to discrimination, immigration, or poverty) [22,24,25], or incorporating important community members (e.g., religious leaders, school counselors) into the treatment process.

Augmentation may be particularly important in the context of Ex-CBT for minoritized youth with anxiety or OCD, as their unique needs were not centered in initial treatment manual development [14,26]. For example, a recent study examining effectiveness of Ex-CBT within a public mental health system serving primarily racially and economically minoritized youth indicated that augmentations, including case management, discussion of cultural and contextual factors, and discussion of general life events, delivered alongside standard Ex-CBT strategies, are producing promising treatment responses [15]. Results demonstrated that youth on Medicaid who completed Ex-CBT treatment were more likely to have received these treatment augmentations compared to those paying for services out of pocket. Yet, treatment rates of response were comparable between youth with and without Medicaid, indicating that treatment effectiveness and successful treatment completion for minoritized youth may have been due, in part, to the incorporation of these augmentations.

Consistent with these findings [15], additional studies suggest that many clinicians are frequently adapting treatment, in the absence of unified guidance, to meet the needs of their clients, and many of these reported adaptations are augmentations to standard protocols [25,27,28]. For example, mental health clinicians in community-based settings frequently report the need to adapt treatment to better fit the cultural values, religious practices, and lived experiences of their clients, particularly when those clients face additional stressors related to their social identities and social determinants of health [28,29].

Given limitations within the cultural adaptation literature, such as overlooking intersectionality and inaccessibility of culturally adapted protocols in usual care [17,30], current emphasis has shifted toward person-centered approaches [17,31,32,33,34,35,36]. Person-centered culturally responsive approaches acknowledge each client holistically to consider not just clinical symptoms, but also salient aspects of their culture and context and how these may be addressed within therapy [36,37,38]. This advances early cultural adaptation protocols, which typically focused on adaptations for individual racial and ethnic groups [17]; while informative, these adaptations may not fully attend to each client’s unique needs. Person-centered culturally responsive care focuses on centering each client’s unique set of salient cultural and contextual factors [36,37]. While conceptually promising, operationalization of person-centered culturally responsive treatment remains limited.

This study takes a practice-based research approach to investigate how clinicians treating minoritized youth with anxiety or OCD within a public mental health setting are addressing client culture and context. Learning from practicing clinicians’ approaches and challenges can help to inform clinical recommendations for more person-centered culturally responsive anxiety and OCD treatment. We examined clinician perspectives on how they aimed to deliver person-centered culturally responsive practices broadly, as well as specific adaptations and augmentations clinicians made to Ex-CBT. Sampling intentionally included clinicians across various settings within a public mental health system to understand how clinicians treating anxious youth (either through Ex-CBT or through alternative treatment approaches) attempt to provide person-centered culturally responsive care. Clinicians in this sample included those working in a specialty anxiety clinic within a public mental health system who have received training and supervision in Ex-CBT, and those working in general community mental health clinics. To our knowledge, this study is the first to broadly examine how clinicians address the culture and context of clients with anxiety and OCD rather than focusing solely on adaptations to a specific treatment protocol. In addition, this study investigates how clinicians incorporate culture and context across their client population, not just with clients from a particular cultural background. This approach can support generalizability, as clinicians within public mental health systems tend to serve youth from varied cultural backgrounds. This study has the potential to inform future efforts to develop more person-centered culturally responsive Ex-CBT for minoritized youth.

## 2. Materials and Methods

### 2.1. Participants

Recruitment occurred as part of a larger study which qualitatively and quantitatively examined clinician, youth client, and caregiver experiences within treatment for anxiety and OCD in community mental health settings [39]. Participants were clinicians (*n* = 16) working within the community behavioral health system in Philadelphia serving youth with anxiety or OCD; eight were working in a specialty anxiety clinic and eight were working in general community mental health clinics. Approximately 6–12 participants are typically sufficient to reach thematic saturation [40]. Therefore, we aimed to recruit at least six clinicians in each group. Additional recruitment was determined to be unnecessary due to saturation being reached during the analysis process. All clinicians worked in clinics that provided Medicaid-funded public mental health services. In Philadelphia, approximately 72% of youth on Medicaid hold racially minoritized identities (52% identify as Black or African American, 23% as Hispanic/Latinx, 6% Asian, and 9% multiple or other races) [41].

Inclusion criteria required that therapists be currently providing treatment for anxiety or OCD symptoms. Participating clinicians had a mean age of 32.19 (SD = 5.87), and 69% of therapists identified as female; 69% identified as White, 25% identified as Asian, and 6% as Black or African American. Clinicians working in the specialty anxiety clinic primarily reported their professional discipline as clinical psychology (*n* = 7), with one clinician reporting their professional discipline as applied behavioral analysis. These clinicians reported their primary therapeutic approach as CBT (*n* = 7). One clinician included family systems and person-centered approaches with CBT, and one clinician reported no theoretical orientation. Clinicians working in general community mental health clinics reported training backgrounds in counseling (*n* = 6) and social work (*n* = 2). These clinicians primarily reported having eclectic therapeutic approaches (*n* = 5) that combined several different approaches, such as CBT, person-centered, strengths-based, and dance/movement therapy. Two clinicians indicated their approach as only person-centered, or strength-based, and one clinician did not respond to this question. See Table 1 for additional participant demographics.

### 2.2. Procedures

All study procedures were approved by the City of Philadelphia IRB (#2021-43). Recruitment began for clinicians working in the specialty anxiety clinic in November 2021 and for clinicians working in general community mental health clinics in December 2021. A brief explanation of the study was sent via email directly to clinicians working in the specialty anxiety clinic and via local listservs that reached clinicians working in general community mental health clinics within the public mental health system. Clinicians completed an electronic consent-to-contact form to indicate their interest in learning more from the research team. Within 48 h, clinicians were contacted to schedule a time for the consent process and interview. Researchers obtained informed consent and conducted semi-structured interviews to gather information regarding clinician experiences addressing their clients’ culture and context. Interviews were digitally recorded with participant permission; clinicians completed a background information survey after the interview. Clinicians received a $50 e-gift card via email for participation. Interviews were developed for the larger study and were guided by the Health Equity Implementation Framework [42] to probe determinants to treatment success and strategies used to support equitable treatment delivery by addressing cultural and contextual factors (see Appendix A for full qualitative interview guide).

Qualitative methods are well-suited for exploring complex phenomena from the participants’ perspectives [43]. Accordingly, we utilized qualitative interviews to explore clinicians’ perspectives and experiences of incorporating culture and context in treatment for youth with anxiety and OCD. Two research coordinators were trained in conducting qualitative interviews through brief didactics, roleplays, and feedback. Didactics included best practices for conducting qualitative interviews. Research coordinators first observed an interview conducted by the second author. The research coordinators then conducted at least two interviews with observation and feedback. Initial interview questions were broad and queried about the clinician’s approach to treatment for youth with anxiety and OCD in their setting. Questions then focused on the clinician’s interpretation of cultural responsiveness (e.g., how they assessed and incorporated culture and context into their treatment plan). If the clinician utilized Ex-CBT, they were then asked about how and why they adapted particular treatment components for their client population. We then probed more specifically about augmentations to address culture and context. Language was modified as needed to examine how clinicians addressed stressors related to their clients’ cultural identities and context, rather than asking about adaptations to treatment, as clinicians in the general community mental health setting expressed confusion about “adapting” treatment, suggesting treatment was always personalized for each client. Interviews were professionally transcribed and uploaded into Dedoose 9.0 software for data management and analysis.

### 2.3. Data Analysis and Theory

An initial coding team from the parent study identified codes, including cultural and contextual factors that impacted care, and one code specific to how clinicians reported addressing cultural and contextual factors. The current study further analyzes data within the code that comprised data on how clinicians reported addressing cultural and contextual factors. The coding team for this study consisted of the first, second, and last authors, who all reviewed excerpts within the code of interest. The first and last authors first engaged in open coding, then further refined codes through a systematic process of double coding subsets of the excerpts until no new codes were identified. The coding team utilized a priori codes related to components of Ex-CBT treatment strategies (e.g., exposure adaptations, cognitive restructuring adaptations), in addition to emergent codes categorizing other ways in which clinicians addressed their anxious and OCD clients’ culture and context.

The coding team iteratively identified new codes, combined existing codes, and clarified definitions of codes until they reached a point of saturation in the data. The point of saturation was determined when no new codes or concepts arose, suggesting that the existing data were sufficiently rich to support holistic interpretation and that additional interviews were unlikely to enhance understanding. The initial coding process resulted in nine preliminary codes: styles of communication, cognitive restructuring adaptations, exposure flexibility/adaptations, tailoring specific techniques to family, assessment of cultural and environmental factors, flexible scope of therapy, pacing, self-awareness and disclosure, and supervision. The coding team met regularly to discuss new insights and revise codes to better reflect emerging meanings. All remaining excerpts were then double coded by the first and last authors. Discrepancies were reviewed collaboratively by the full coding team (first, second, and last authors), with the second author serving as a tiebreaker when needed. Following code finalization, the team conducted thematic analysis to identify patterns of meaning across and within the codes. This involved examining relationships among codes, grouping related concepts, and organizing them into seven themes that captured the central processes and principles reflected in participants’ responses. The authors also reviewed the extent to which themes were referenced across clinician groups (i.e., those working in a specialty anxiety clinic and those working in general community mental health clinics).

### 2.4. Positionality

The first, second, and last authors were engaged in thematic analysis, which was influenced broadly by our own backgrounds, lived experience, and programs of research. We acknowledge our positionality as a team identifying as cis-gender female and representing various racial backgrounds (i.e., White and Latina) with a primarily cognitive–behavioral theoretical orientation. We are committed to community-based research and enhancing the cultural responsiveness and accessibility of evidence-based practices. Our coding team encompassed a range of perspectives, including clinicians and a doctoral graduate student. The first and second authors bring perspectives from both lived and professional experiences with minoritized identities. The last author brings insight as a clinician working within a specialty mental health clinic within a community setting. Our data interpretation benefited from diverse viewpoints expressed in group discussions.

## 3. Results

Clinicians described incorporating culture and context through adapting existing Ex-CBT techniques, augmenting treatment with strategies not traditionally included within Ex-CBT protocols to meet client needs, and attending to the overall treatment process. We organized themes within two overarching therapeutic phases: Culturally responsive assessment, case conceptualization, and treatment planning; and Culturally responsive treatment delivery and process. Within these phases, we identified seven themes and seven subthemes that illustrate how clinicians aim to provide culturally responsive care for youth with anxiety and OCD (see Table 2 for summary of themes, subthemes, and corresponding clinical implications). Most themes were endorsed across clinician groups, but those focused on adaptations and augmentations to Ex-CBT emerged more prominently among clinicians working in the specialty anxiety clinic (see Table 3 for themes, subthemes, and illustrative quotes).

### 3.1. Therapeutic Phase: Culturally Responsive Assessment, Case Conceptualization, and Treatment Planning

Researchers identified two themes within the phase of culturally responsive assessment, case conceptualization, and treatment planning; Theme 1: developing a culturally informed case conceptualization of the client and their anxious distress; and Theme 2: taking a holistic, systems-informed approach to treatment planning.

#### 3.1.1. Theme 1: Developing a Culturally Informed Conceptualization of the Client and Their Anxious Distress

All clinicians highlighted the importance of developing a holistic understanding of youth clients’ presenting concerns within the culture (e.g., salient social identities, beliefs about mental health and treatment, values) and context (e.g., stressors and strengths related to their social identities and lived environment) of both the client and their family. Subthemes included (1a) proactively and continually assessing cultural context, and (1b) understanding family cultural norms and expectations and their intersection with anxiety and OCD.

1a. Proactively and Continuously Assessing Cultural Context

Clinicians in both settings emphasized the importance of assessing salient cultural and contextual factors both at the outset and throughout the course of treatment. This step was described as essential for helping families feel heard and understood, and for enabling clinicians to develop accurate and responsive case conceptualizations. One clinician described gathering cultural information during initial sessions by creating a family genogram and exploring past experiences with mental health care while also leaving space in later sessions for clients to share cultural identities and traditions when they felt ready. The clinician noted, “just always asking how they would identify … giving them the opportunity to disclose that if they feel comfortable … and if they don’t feel comfortable disclosing right away … circle back through it later” (General Community Mental Health Clinic, Clinician 8).

Several clinicians reflected on how their practices have evolved to place greater emphasis on proactive assessment of cultural and contextual factors. One clinician noted that early in their career, cultural issues often emerged only in response to challenges or treatment barriers:

*“I don’t think in [the clinic] … we did a lot of, like, specific questioning about cultural values. It was more as it came up in the form of barriers … for example, there’s a case of a family that was Muslim and so there were specific views with different members of the family about mental health treatment … specific diagnoses, and medication. … And so it was a little bit, like … reactive, but maybe could have been more proactive.”*

(Specialty Anxiety Clinic, Clinician 3)

In contrast, another clinician shared that their, “progression has been from…thinking that the information I got at the front end was what I needed, to recognizing that this is actually really an ongoing process” (Specialty Anxiety Clinic, Clinician 4). These perspectives reflected a developmental trajectory in clinician practice, from viewing culture as static or peripheral to client care, to engaging with it as a dynamic process throughout the course of care.

Several clinicians emphasized the importance of clinicians, rather than clients, initiating conversations about culture, identity, and societal events, particularly given the inherent power dynamics in clinical relationships. For example, one client shared:

*“I don’t really shy away from those topics … if I have somebody that’s sitting in front of me, that’s either a different race, or a different gender or different religion … there’s obviously a dynamic that goes into that.”*

(General Community Mental Health Clinic, Clinician 14)

At the same time, some clinicians discussed hesitancy to bring up cultural or contextual stressors. One clinician explained their approach to acknowledging the potential impact of current events on clients, but noted they might not raise these topics unprompted:

*“Like, if racial protests are going on, I like to ask how clients are handling it, what their thoughts are on it … if they had ever experienced racial discrimination … I don’t think I go out of my way to talk about it if they haven’t mentioned anything, but if they ever allude to the fact that they’ve experienced discrimination, I do make time for it.”*

(Specialty Anxiety Clinic, Clinician 5)

In summary, clinicians’ perspectives demonstrated an awareness of the importance of continually assessing the impact of culture and context on their clients’ experiences of mental health, yet there was variability in clinicians’ comfort levels and knowledge of how to do so.

1b. Understanding Family Cultural Norms and Expectations and their Intersection with Anxiety and OCD

Clinicians in both groups emphasized how, with children, it is important to assess not only the child’s culture and context, but also that of their family and community systems. Clinicians highlighted challenges related to differences in acculturation, beliefs about mental health, and values related to social identity, especially religious values. As described by one clinician:

*“When you’re working with children …the child’s cultural identity is often very different than the family’s, than the parents,’ or even siblings’. So just holding space for different members of the family system to have very different identities and be really open to exploring that.”*

(Specialty Anxiety Clinic, Clinician 2)

Clinicians emphasized the importance of remaining open and curious about family roles and expectations that may differ from their own cultural frameworks. Rather than pathologizing what initially appears unfamiliar, several clinicians described efforts to explore cultural norms with humility. One clinician reflected on this process when working with a mother who assumed a primary caregiving role in her family. Although the clinician initially perceived the mother as overburdened due to lack of partner support, they paused to reflect and reframe, “I was even challenged with … asking the mom, what is normative in your family? What are the roles and expectations …? Is it that you are tasked with the caregiving and that is what is normative for … your family? … Ideally, the thing you get to do is ask questions about it and not assume that there’s something wrong happening just because it may seem off to me” (General Community Mental Health Clinic, Clinician 15).

Clinicians also discussed cultural norms as they related to conceptualizing anxiety and OCD more specifically. Clinicians often highlighted challenges with determining the extent to which intrusive thoughts or fears were normative given the family’s cultural context or if they were true obsessions. In many instances, clinicians reported that conceptualizing anxiety and OCD within cultural norms felt murky and highly nuanced. For example, multiple clinicians discussed the challenge of disentangling spiritual beliefs from unrealistic anxious thoughts and obsessions, and the importance of in-depth cultural understanding and collaborative conversations with clients, families, and spiritual leaders to carefully select culturally appropriate Ex-CBT targets. One clinician shared:

*”If we’re talking about a kid who has OCD religious obsessions … I would really want to know… what do you believe? … I would … involve religious leaders in trying to determine what are … some exposures we can do that might violate what would typically be done? And … trying to recruit people who can provide me with additional information… and identify how that cultural experience intersects with the anxiety and OCD experience.”*

(Specialty Anxiety Clinic, Clinician 4)

Another clinician shared about a client with intrusive thoughts related to her sexuality:

*“And understanding the family system, father was [really involved] in the church, and was this an intrusive thought that she was a lesbian, or was it [a] feeling of distress because she might be a lesbian? And how did that intersect with her religion?”*

(Specialty Anxiety Clinic, Clinician 9)

Overall, clinicians emphasized understanding the client’s values while noting how these may differ from family or community values, as well as conceptualizing client fears within the context of cultural norms and expectations. Rather than dismissing fears as irrational, the clinicians attempted to understand the nuances of clients’ fears within their cultural context.

#### 3.1.2. Theme 2: Taking a Holistic, Systems-Informed Approach to Treatment Planning

Clinicians across settings emphasized that many youth clients faced substantial contextual and social identity-related stressors, such as housing instability, unsafe neighborhoods, immigration-related fears, and limited access to transportation or healthcare. These stressors often shaped not only the clients’ presenting concerns but also their ability to consistently engage in psychotherapy. Clinicians described augmenting treatment or broadening treatment foci beyond individual-level symptom reduction to take a more holistic, systems-informed approach. Subthemes included (2a) evaluating appropriateness of Ex-CBT techniques within clients’ environmental context, and (2b) addressing environmental stressors and structural barriers.

2a. Determining the Appropriateness of Ex-CBT Techniques within Clients’ Environmental Context

Clinicians described challenges in distinguishing fears that were disproportionate or unrealistic, and thus amenable to traditional Ex-CBT, from those that were normative within the client’s culture (e.g., religious beliefs or rituals) or context (environmental stressors such as food/housing insecurity, community violence) and, therefore, more appropriately targeted with non-exposure strategies (e.g., problem solving, case management, obtaining educational supports). Clinicians discussed the nuance of “accepting that this individual is living in this environment and these anxieties that provide safety… are there for a purpose[,]” (General Community Mental Health Clinic, Clinician 14) and recognizing that while avoidance and hypervigilance may be protective in certain settings, or may have been in the past, these behaviors may be getting in the way in other settings. One clinician shared how they validated that the client’s anxiety has served them in certain ways and may not serve them in others:

*“I get why you have to do this in this specific environment, but let’s … see if it works in these other environments, too. Do these certain things work for you when you’re at school? … And then we get to work on the anxiety in the school setting while they still could keep up their protective factors when they go into these places where they need to have their guard up.”*

(General Community Mental Health Clinic, Clinician 14)

Another clinician described this decision-making process when supporting a client who feared the deportation of their parents:

*“Trying to tease apart the realistic nature of the fear versus, like, is this something we should target with exposures, is this something we target with problem solving? … I had a patient who was worried about her parents being deported … and I was, like, that’s a realistic fear, based on what was happening at that time. And so, we had conversations about it that… didn’t use a typical CBT approach … I definitely made adjustments to treatment to help her just cope with the fact that those thoughts are just really big and scary.”*

(Specialty Anxiety Clinic, Clinician 1)

Clinicians acknowledged that even when fears are rooted in real environmental risks, clients may still experience significant anxiety and disproportionate avoidance that interferes with functioning. In such cases, clinicians described trying to navigate a delicate line between validating legitimate concerns and supporting the development of adaptive coping and exposure-based strategies. One clinician described working with a client who was afraid to take public transportation due to fears of being attacked; the clinician described validating the client’s lived experiences and collaborating with them to identify strategies for staying safe, while also reducing maladaptive avoidance of public transportation:

*“She always felt like, on the train, that if somebody moved their bag, that they would have a weapon in there … that she would be a victim of violent crime at any time … And I didn’t want to push her and discount some of the real problems that women face in male violence, right? So, we… talked about why it might still be important [to take the train], what we’re going to keep an eye on … texting her parents if she feels uncomfortable, taking a phone call, carrying mace … things that were safety planning, rather than safety behaviors.”*

(Specialty Anxiety Clinic, Clinician 5)

Overall, clinicians emphasized the importance of understanding how their clients’ life circumstances influenced whether fears should be targeted with exposure or with non-exposure strategies. Clinicians described working to avoid pathologizing client concerns that were grounded in contextual stressors, while still helping clients engage with treatment goals.

2b. Addressing Environmental Stressors and Structural Barriers

Many clinicians underscored the importance of first addressing clients’ basic needs (e.g., food, housing, and safety) as a prerequisite for engaging in psychotherapy. When these needs were unmet, clinicians described shifting their clinical priorities. As one clinician stated:

*“A lot of my clients have difficulties with transportation, neighborhood safety … they’re dealing with very real stressors. They’re hungry, they’re trying to figure out where they’re going to live … they can’t focus on more abstract concepts. They’re focused on just surviving. Sessions usually get brought back to those basic needs … allowing that flow in sessions. Because if those things aren’t satisfied or are causing immense stress, then the work we do … isn’t going to be as effective.”*

(General Community Mental Health Clinic, Clinician 14)

Clinicians discussed “the systems work that often needs to happen … what other supports the family might need—case management or collaboration or advocacy” (Specialty Anxiety Clinic, Clinician 2). Clinicians also described seeing it as part of their role to support clients’ basic needs by coordinating with multiple systems, including schools, healthcare providers, caseworkers, and community organizations, to effectively support youth and families. Clinicians described advocating for school accommodations and consulting with primary care or psychiatric providers to discuss clients’ needs. For many clinicians, this work was essential to address structural barriers and bolster treatment adherence. As one clinician explained:

*“…seeing my role as not just individual therapy with a child but really considering the big picture and what this family needs … that either I can provide, or I can connect them with someone to provide. So, from a sort of hierarchy and needs perspective, if the family is worried about the parent losing their job … understandably, treatment’s going to fall lower on the priority list.”*

(Specialty Anxiety Clinic, Clinician 2)

Clinicians also highlighted the need to link clients with specific community supports, especially for families navigating systemic exclusion. As illustrated by one clinician:

*“First, I’m trying to see … how I might be helpful with improving [a client’s] immigrant status … how to help them feel more safe. Many of them do not have insurance … legal income … they do not feel themselves part of this country… and it increases the anxiety, the depression, the other mental health problems.”*

(General Community Mental Health Clinic, Clinician 11)

Overall, clinicians described augmenting traditional treatment with advocacy and community collaboration, particularly for families facing negative social determinants of health. Clinicians emphasized that their clients needed more than the standard individual psychotherapy, and that it may be within the clinician’s role to collaborate with multiple systems to ensure their clients’ needs are fully supported, even beyond what traditional Ex-CBT provides.

### 3.2. Therapeutic Phase: Culturally Responsive Treatment Delivery and Process

Researchers identified five themes within the phase of treatment delivery and process; Theme 3: aligning Ex-CBT techniques with client values and cultural traditions; Theme 4: incorporating social identity into exposure planning; Theme 5: engaging in collaborative decision-making and flexibility; Theme 6: building trust; and Theme 7: engaging in self-reflection as a tool to improve cultural responsiveness. Themes 3 and 4 were discussed only by clinicians working in the specialty anxiety clinic, and all other themes were discussed by both clinician groups.

#### 3.2.1. Theme 3: Aligning Ex-CBT Techniques with Client Values and Cultural Traditions

Clinicians within the specialty anxiety clinic setting often reflected on how core aspects of CBT, such as psychoeducation, cognitive restructuring, and exposure work, may rest on implicit assumptions about individualism, emotional expression, and what constitutes adaptive functioning. As such, many clinicians from the specialty anxiety clinic setting described the need to pause and examine how Ex-CBT content and delivery could better resonate with clients’ lived experiences and belief systems. This resulted in intentional adaptations to align Ex-CBT with the cultural values, worldviews, and communication preferences of clients and their families.

Several clinicians working in the specialty anxiety clinic emphasized the importance of adapting how content was delivered, in addition to considering what content was delivered. For example, one clinician described the value of adapting psychoeducation to match cultural norms around authority and communication:

*“So, there’s the actual content that I need to deliver … but potentially the way that I communicate it with my style … or who is actually delivering the message is going to be important … I might consider bringing in a religious leader or somebody who can explain it in a way that’s … aligned with their cultural principles.”*

(Specialty Anxiety Clinic, Clinician 7)

Other clinicians noted the importance of tailoring how they presented treatment rationales, such as emphasizing research-based effectiveness versus personal clinical experience, depending on the family’s comfort and trust in research. One clinician explained:

*“There are families I work with where it’s really comforting for them to hear that the research suggests this is super effective* *. And for other families, there’s a lot of understandable suspicion about research. And what they want to hear really is like …I’ve seen this be helpful for kids like yours. So just even the way I communicate messages about effectiveness … often needs to be adapted to what the family is comfortable with and what they value.”*

(Specialty Anxiety Clinic, Clinician 2)

Clinicians also reflected on how cultural values, parenting practices, and intersecting social identities shaped how families engaged with treatment. Some clinicians shared moments of missed opportunities where deeper exploration of family values could have enhanced treatment alignment. As one clinician described:

*“I did not adequately assess and consider their parenting beliefs based on their generation, their race, dad’s military background, the neighborhood where they lived … I don’t think that I took enough time to really pause and ask them, like, what are your parenting beliefs? How could this fit with what I’m suggesting?”*

(Specialty Anxiety Clinic, Clinician 4)

This value-alignment extended to the design and implementation of exposure tasks, with therapists noting the need to consider cultural norms, logistical realities, and family preferences in exposure practice. As one clinician noted:

*“It’s more about just making sure that the exposures themselves fit in terms of the child’s needs, but also the family’s values, preferences, attitudes, capabilities,* *logistics—all those pieces.”*

(Specialty Anxiety Clinic, Clinician 2)

Cognitive restructuring was noted as an area requiring uniquely careful attention due to its embedded assumptions about what constitutes rational or adaptive thinking. For example, one clinician explained:

*“Our idea of what thoughts are right and wrong comes from our cultural lens … I just need to be really careful that [my framing] actually is aligned with the reality that they experience.”*

(Specialty Anxiety Clinic, Clinician 4)

Clinicians emphasized the need to avoid imposing their own cultural framework when labeling thoughts as distorted or inaccurate. One clinician described:

*“I really have to be careful … I have to make sure that some of the assumptions I’m using when I do flexible thinking, are taking more of a soft, universal approach. Not all cultures believe that you’re allowed to take a step back from school just to work on your mental health … I think that is the most adjustments I have to make.”*

(Specialty Anxiety Clinic, Clinician 5)

Clinician also described approaching treatment adaptation in a collaborative, humble manner. As one clinician shared:

*“[It’s] not just about, hey, I have CBT, I had the [exposure and response prevention], and I’m sorry that your religious or personal beliefs don’t align with that. It’s just what we’re doing. [Instead, asking] how can we align, and how can I understand to help you be the best you based upon your needs and really understanding your culture?”*

(Specialty Anxiety Clinic, Clinician 9)

Ultimately, clinicians described culturally aligning Ex-CBT techniques through adapting how they communicate psychoeducation materials including treatment rationales and the concept of thoughts as rational or irrational. Clinicians described treatment adaptation as an ongoing process requiring humility and collaboration.

#### 3.2.2. Theme 4: Incorporating Social Identity into Exposure Planning

As noted above, even once it was determined that Ex-CBT was appropriate, clinicians working in the specialty anxiety clinic reported the need to recognize how clients’ social identities may influence their anxiety and OCD symptoms and, therefore, could be incorporated into the exposure planning. One clinician shared their experience of trying to develop exposures that would target their client’s anxiety without putting the client in a position to experience additional emotional harm associated with identity-based discrimination:

*“[The client] told me that he had come out as gay and his family … and his community, all kind of rejected it … A lot of the things he would want to do to… be his authentic self were not safe social anxiety exposures for him … There were unrealistic fears related to social anxiety, but then there were also realistic fears related to discrimination, and how do we make sure that our exposures are only targeting the unrealistic fears but not fears that are valid and keeping him safe?”*

(Specialty Anxiety Clinic, Clinician 2)

Another clinician reported recognizing, during the exposure practice, that experiences of racism and discrimination were making certain exposures more difficult for their client who identified as African American. Once the clinician understood this about their client, they were able to discuss these situations more openly and develop more appropriate exposures:

*“We adjusted to do other public exposures that didn’t… [make] her feel additionally uncomfortable. And I also rethought that … maybe this place is actually higher on her hierarchy and it’s unfair to put her in such a difficult place … So, I did not continue to make her go to a place that she felt racially and socioeconomically uncomfortable, but I didn’t stop doing exposures. We would instead go to … more diverse places, like we would go to the hospital across the street … because it seemed a little more balanced from SES.”*

(Specialty Anxiety Clinic, Clinician 5)

Overall, clinicians recognized that their clients’ social identities and related stressors often influenced not jonly their experiences of anxiety, but also the effectiveness and relevance of their exposure hierarchies. Importantly, while clinicians desired to respect their clients’ lived experiences and facilitate values aligned gradual exposure, clinicians shared that it was only after trial and error that they recognized how their clients’ identities played a role in their anxiety and the exposures they conducted.

#### 3.2.3. Theme 5: Engaging in Collaborative Decision-Making and Flexibility

Clinicians from both groups, but especially those from general community clinic settings who were more likely to report professional disciplines of social work and counseling, emphasized the importance of acknowledging the client as the expert on their own experience and being flexible in session to follow the client’s lead on how best to incorporate relevant cultural and contextual factors. Many clinicians noted that these approaches were especially important for families who have faced marginalization and negative experiences within the healthcare system to feel empowered to be part of the treatment team and make decisions about their care. Clinicians highlighted the importance of recognizing that “clients are the experts of their own life” (General Community Mental Health Clinic, Clinician 8). One clinician shared how they approached collaboration throughout treatment:

*“I would … ask them, like, what do you actually want to work on? So, at least it gives them the autonomy to … decide … And then, usually … I’ll bring up the techniques I used to work with clients in the past. But I also will tell them, sometimes it works with people, sometimes it doesn’t … because everyone’s different … So, I would ask them, how do you feel if you try this? … So, that’s how we end up doing the treatment plan.”*

(General Community Mental Health Clinic, Clinician 10)

Another clinician shared their experience with a client who had a pregnancy scare and how they shifted to meet the client’s immediate needs:

*“We’re not [doing] exposures today. I know you have panic attacks. We’ll get back to that … how can I support you? What resources and steps do you need? … I just was like full-on, what do you need from me?”*

(Specialty Anxiety Clinic, Clinician 1)

Clinicians discussed collaboration across all aspects of treatment. One clinician highlighted the importance of “approaching everything with an attitude of curiosity and not… assuming that a certain strategy or approach is going to work for a family or system, but really trying to … be really exploratory, both with respect to families’ resources and capabilities, and values and attitudes. And … continually modeling … [a] willingness to be collaborative and shift” (Specialty Anxiety Clinic, Clinician 2).

In summary, clinicians highlighted maintaining an open, flexible approach to treatment that welcomes client input and feedback. Clinicians further emphasized empowering clients as active members of the treatment team who have important input regarding what should be included in their treatment. 

#### 3.2.4. Theme 6: Building Trust

Clinicians in both settings shared a common perception that many of the youth seen in community mental health settings, commonly youth with minoritized identities, have had negative experiences in the mental health system, leading to mistrust. Clinicians shared how they addressed this by intentionally building trust in the therapeutic relationship prior to engaging in some treatment techniques and throughout the treatment process. Subthemes included (6a) building trust through self-disclosure, (6b) building trust through recognition and validation of negative experiences, and (6c) building trust takes time.

6a. Building Trust Through Self-Disclosure

Clinicians discussed the importance of strategic self-disclosure to build trust and rapport with clients, particularly those from minoritized backgrounds. Clinicians most frequently emphasized self-disclosure of social identity, both those shared and unshared with clients’ identities, but also noted disclosure of shared mental health or contextual stressors. Several clinicians described how explicitly naming differences in identity and lived experience could open the door to trust and foster a more collaborative therapeutic relationship. As one clinician explained:

*“Just naming the differences and naming that you might not understand their experiences because you haven’t gone through it specifically, but you’re here to try and understand … And I found that just by doing that, it builds a lot of trust and just understanding.”*

(General Community Mental Health Clinic, Clinician 14)

In contrast, several clinicians with minoritized identities explained that their shared identity helped clients feel more comfortable discussing social identity-related stressors. For example, a Black clinician shared that their identity helped their clients feel more comfortable discussing experiences of discrimination:

*“When clients brought up issues of racial discrimination, I feel like it’s something that needs to be talked about. And I think that … oftentimes a lot of people of color or just people who are oppressed in general don’t really feel like they have the space to speak to about it. And I think that’s one of the ways in which … being a Black woman is helpful, because if that is something people are struggling with, they feel comfortable talking about it.”*

(General Community Mental Health Clinic, Clinician 15)

Similarly, a clinician who identified as gay shared that disclosing their sexual orientation helped a client feel more at ease and avoid the emotional labor of explaining aspects of their identity:

*“I am gay too, so I have a decent understanding of what the community is about and the basic terms … And it immediately clicked with him … it seemed like he felt thankful that he didn’t have to educate me … I felt a shift in the rapport immediately.”*

(General Community Mental Health Clinic, Clinician 14)

Despite recognizing the power of identity-based self-disclosure, several clinicians noted uncertainty about when and how to engage in tself-disclosure. Furthermore, clinicians highlighted a lack of formal training in utilizing self-disclosure. As one clinician shared:

*“I’m always navigating … how much [self-disclosure] do I bring into the space and when and how? And honestly, it’s a place where I would love to get more training. I think it’s still a growth area for me.”*

(Specialty Clinic, Clinician 2)

Another clinician noted the importance of self-disclosure of identity to help foster discussion of clients’ identities and facilitate the adaptation process as a whole:

*“I think the thing that really stands out as … missing from my training was learning—which I’ve just sort of done trial and error—how to self-disclose, and how to talk about my own identity and ask about clients’ identities. Because I actually think that once that’s out on the table … the adapting treatment part comes more easily and naturally.”*

(Specialty Anxiety Clinic, Clinician 4)

Overall, judicious self-disclosure emerged as a key relational strategy for building trust with clients, particularly in addressing social identity and contextual stressors. Importantly, clinicians described using disclosure not to center themselves, but to create space for shared understanding and to emphasize psychological safety in the therapeutic space. While many clinicians felt confident in the power of self-disclosure, they also highlighted a clear need for more guidance and training on how to navigate these conversations ethically and effectively.

6b. Building Trust Through Recognition and Validation of Negative Experiences

Clinicians emphasized that, given historical and current mistreatment of minoritized communities, acknowledging and validating mistreatment can build trust and rapport. For example, one clinician who provided in-home services described the importance of directly addressing families’ prior encounters with the child welfare system:

*“There’s a lot of associations that are unique to our situation [such as Child Protective Services] … I like to be very explicit about, like, what experiences have you had with these other agencies, like, what does this feel like … to have someone come into your house? … Being able to just identify what maybe negative experiences have happened in the past that could be associated with our work in the present.”*

(Specialty Anxiety Clinic, Clinician 6)

Clinicians also spoke to the importance of validating the reality of clients’ lived experiences—especially those involving discrimination, marginalization, or systemic barriers—even when such experiences may not be “solvable” within treatment. One therapist reflected on how this can feel counterintuitive within a cognitive–behavioral framework:

*“It’s tough because I think that CBT therapists … want to be really active. And there’s not a solution for [systemic oppression], right? … Sometimes the best we can do is validate and not try to solve it, and certainly not trying to challenge a cognition that’s an accurate one. And … cultivate space, that this is an okay thing to talk about, and it’s not an acceptable thing to be happening and your experience is real. And that’s it … You can’t say, you need to think about this differently, you need to do something differently … And I think as a CBT therapist, that feels super uncomfortable for me, and I have to just sit with that discomfort.”*

(Specialty Anxiety Clinic, Clinician 2)

Clinicians recognized that validating clients’ negative experiences within systems was essential for building trust and rapport. Clinicians described that building trust often required holding space without seeking to problem-solve or reframe, particularly when clients’ beliefs or fears were grounded in lived experiences of harm.

6c. Building Trust Takes Time

Clinicians repeatedly emphasized that building trust—particularly with youth from minoritized backgrounds—required time, flexibility, and attunement to individual client needs. Many clinicians shared that they intentionally slowed the pace of treatment to prioritize rapport-building before introducing more directive therapeutic strategies. One clinician reflected on a case where insufficient time for rapport-building may have disrupted treatment progress:

*“[Slowing] the pacing of treatment, and potentially spending a lot more time on psychoeducation and rapport-building … is kind of what I wish I had done with a patient, that it didn’t necessarily go super well … We probably tried to move too quickly for what this patient was ready for … both from an individual perspective, but also their cultural beliefs about anxiety and about therapy and our different identities. I think we needed more time to build up rapport and trust before jumping in as much to active intervention.”*

(Specialty Anxiety Clinic, Clinician 3)

At the same time, clinicians acknowledged that trust may not always develop, especially when working with youth who have experienced significant trauma or institutional betrayal. One therapist who worked with clients in the child welfare system reflected the following:

*“One of the more challenging parts of the population that … I … currently work with, being in the child welfare system … our kids have had traumatic experiences and absence of… family members … Some kids have lived on the streets for the entirety of their lives and have no family members at their disposal. So, establishing trust is an ongoing thing and it’s a constant challenge … And I’m aware that, due to the nature of the trauma that our kids have experienced in their lives, sometimes a trusting relationship … may take a prolonged period of time, or sometimes it may happen instantly … or never.”*

(General Community Mental Health Clinic, Clinician 12)

Other clinicians noted the nuance of adjusting the pace of treatment based on individual needs, noting that some clients may require extended rapport-building before treatment can begin, while others may develop trust only after experiencing the benefits of therapy. As one clinician described:

*“There’s clients where rapport-building comes with just spending more time getting to know them. And then there’s other clients that are in real distress, and they’re not going to trust you until you show you can help them … I just always had to keep in the back of my head: does this client need more time with me … or do we need to just move forward, and the trust will come?”*

(Specialty Anxiety Clinic, Clinician 2)

Clinicians underscored that building trust is an individualized process, especially for youth with complex histories and minoritized identities. While some clients benefit from an extended period of relationship-building and psychoeducation, others may come to trust their clinician only after experiencing tangible benefits from treatment.

#### 3.2.5. Theme 7: Engaging in Self-Reflection as a Tool to Improve Cultural Responsiveness

Across both clinic settings, clinicians emphasized the ongoing process of self-reflection and learning as central to being able to address their clients’ culture and context in therapy. Many clinicians described efforts to improve their ability to elicit clients’ cultural and contextual experiences and incorporate this information into case conceptualization and treatment planning. Clinicians, particularly those with privileged identities, noted that this work often began with confronting their own discomfort or lack of awareness.

Several White clinicians described how engaging in self-reflection helped them recognize how their privileged identities shaped their initial hesitation to discuss culture and identity. As one therapist shared:

*“I found that as a White person … or as a male… and all those privileged identities that I hold, that the first instinct is to shy away from those discussions … but I found that that’s not helpful to anybody, including myself. So really throwing myself into those discussions allows the conversation to start. So … reflecting on my own identities and how they might show up in session … has been really helpful at either developing rapport or having people feel understood.”*

(General Community Mental Health Clinic, Clinician 14)

Clinicians also spoke about the importance of clinical humility and the willingness to engage imperfectly rather than avoid cultural conversations altogether. As one therapist reflected:

*“I think it is a core aspect of providing ethical, effective clinical care to have those conversations, to be willing to mess up sometimes in the interest of serving the clients and honoring, respecting their backgrounds and tailoring treatment to meet their unique needs.”*

(Specialty Anxiety Clinic, Clinician 4)

Several clinicians noted initial discomfort or uncertainty about how to broach cultural topics in ways that felt respectful and open-ended. One clinician described how structured tools, such as the Cultural Formulation Interview, have helped build their confidence and skill:

*“I used to have a lot of trouble asking about those things without pinning certain values or identities on a client … So, I will pull [the cultural assessment] up … to remind myself of phrasings and wording that I can use that doesn’t assume anything and allows the client to kind of go any direction they want.”*

(Specialty Anxiety Clinic, Clinician 5)

Other clinicians expressed a desire for more guidance in understanding how culture and context can shape case conceptualization and treatment planning. One clinician emphasized the value of incorporating cultural considerations into supervision and clinical decision-making:

*“Trying to anticipate potential conflicts or cultural issues in supervision would be helpful in case conceptualization, particularly, but also using that to then guide, okay, now you’re planning for this phase of treatment. So, let’s think about what might come up for this family in this context.”*

(Specialty Anxiety Clinic, Clinician 3)

Together, these reflections highlight clinicians’ recognition that culturally responsive practice requires continual reflexivity and willingness to learn. Clinicians emphasized the need for structured support and tools to better integrate cultural awareness into all aspects of clinical care. By reflecting on their own identities and the power dynamics present in cross-cultural therapeutic relationships, many clinicians reported a growing willingness to engage in meaningful, if sometimes uncomfortable, conversations about culture; they acknowledged that avoiding these discussions could undermine the therapeutic relationship, whereas approaching them with openness and humility could build trust and support more culturally responsive and effective care.

## 4. Discussion

Ex-CBT is the current gold-standard treatment for anxiety and OCD; however, its applicability to minoritized youth remains in question as they are not represented in initial efficacy trials, and cultural and contextual factors are not systematically integrated into treatment protocols [14]. To our knowledge, this is the first study to engage in practice-based research to understand the ways in which clinicians address culture and context to better serve clients with anxiety and OCD. This study supports the previous literature, as clinicians shared the need to make adaptations to attend to their clients’ culture and context [27,28,29]. In addition, this study goes beyond the previous literature to describe specific considerations for youth with anxiety and OCD. Clinicians shared experiences of Ex-CBT-specific adaptations and augmentations that address cultural and contextual factors, and process-based approaches to support person-centered culturally responsive carefor minoritized youth with anxiety and OCD.

Importantly, synthesis of clinician reflections resulted in clinical considerations and concrete strategies that hold potential to guide person-centered culturally responsive treatment for youth with anxiety and OCD to be tested in future studies. Qualitative feedback from clinicians coalesced around two major components of person-centered culturally responsive care for youth with anxiety and OCD. First, clinicians described underlying therapeutic process factors agnostic to any specific evidence-based treatment protocol (e.g., cultural assessment, collaborative decision-making, discussing identity, pacing, and trust), that they viewed as essential to delivering culturally responsive care. Second, clinicians using the Ex-CBT protocol highlighted the need for thoughtful application of Ex-CBT principles through adaptation, augmentation, and consideration of potential risks of misapplying Ex-CBT principles, while also ensuring that Ex-CBT’s potential to alleviate youth distress associated with maladaptive avoidance was realized.

### 4.1. Therapeutic Process Factors to Facilitate Person-Centered Culturally Responsive Care

Both clinician accounts and the emerging literature highlight cultural assessment as a critical early and ongoing step for delivering person-centered culturally responsive care. Preliminary evidence indicates that cultural assessment can support many aspects of client care, (e.g., engagement, feeling understood, clinician satisfaction, and differential diagnoses) across various therapeutic approaches [36,45,46]. Clinicians emphasized the value of assessing culture not only at the individual level but also within the family system. Youth and caregivers often differ in levels of acculturation, views on mental health, and treatment goals [47,48,49,50], all of which are factors that can shape treatment preferences. Cultural assessment may help families navigate these differences to establish shared treatment goals and improve treatment engagement and success [51,52,53]. However, cultural assessment has not been a part of traditional diagnostic assessments [54,55], and structured cultural assessment tools have only recently been developed, leaving clinicians with little guidance on how to broach conversations about clients’ culture and context. More work is needed to train clinicians in how to engage in cultural assessment and examine the ways in which cultural assessment can influence treatment planning and clinical outcomes [45].

Clinicians in this study also described facilitating person-centered culturally responsive care through collaborative decision-making and building trust. These practices are consistent with the current literature demonstrating that collaborative decision-making is a powerful tool for improving client engagement and treatment fit [56,57], and that it may be particularly useful when there are differing perspectives and cultural values within the family system [57,58]. Clinicians in this study described collaborative decision-making as a way to show respect for clients’ and families’ perspectives, which in turn strengthened trust. These practices align with research demonstrating that mutual respect and collaboration can result in stronger therapeutic relationships and trust [59,60].

Clinicians also specifically highlighted the need to attend to the therapeutic relationship and build trust with their minoritized clients who may have had more negative experiences within the mental health system. This is particularly important as the therapeutic relationship is a strong predictor of treatment engagement and outcomes [61,62], and clients from minoritized groups disproportionately experience poor therapeutic relationships compared to non-minoritized clients [63,64]. Clinicians discussed building trust through pacing (i.e., slowing down or speeding up the therapeutic content depending on their clients’ needs), validating their clients lived experiences, and engaging in discussion of identity. Of note, several clinicians discussed the importance of clinicians initiating conversations about identity and engaging in judicious self-disclosure of their own salient identities. Simultaneously, some clinicians in our sample expressed discomfort discussing identity, even though these discussions are central to topics of clients’ culture and context; this discomfort discussing identity is consistent with the previous literature demonstrating that some clinicians express a lack of confidence in broaching topics of identity and may avoid doing so altogether [65,66,67,68]. As such, future efforts should prioritize the development of Ex-CBT training and supervision models that equip clinicians with skills (e.g., cultural humility, discussing social identity, collaborative decision-making, ecological systems awareness) to support more culturally responsive treatment planning and positive treatment outcomes.

### 4.2. Thoughtful Application of Ex-CBT Principles Through Adaptation and Augmentation

When using Ex-CBT with clients who held minoritized identities, many clinicians working in the specialty anxiety clinic described struggling to conceptualize their clients’ fears within the Ex-CBT framework, particularly in distinguishing between “realistic” and “unrealistic” fears. Clinicians also discussed the potential harm that could come from attempting to restructure accurate thoughts, especially those associated with environmental and identity-related stressors (e.g., racism, discrimination, immigration status, poverty, community violence). One illustrative example emerged in a clinician’s account of working with a client who feared using public transportation. While traditional Ex-CBT might conceptualize this client’s avoidance and associated safety behaviors as maladaptive, for a youth client living in a neighborhood with frequent community violence, fear of being harmed on public transportation may not be irrational; however, this fear and associated avoidance may still interfere with the client’s ability to engage in important daily activities (e.g., going to school, participating in social activities). In the case described by this clinician, there was actual risk, and an adaptive nature of the fear, yet complete avoidance was maladaptive for this client. The clinician noted working with the client’s family to determine normative behavior (e.g., taking the bus alone), and to develop a safety plan (i.e., steps to take if the client feels unsafe, how to assess for safety) prior to engaging in exposure. Overall, clinicians cautioned against conceptualizing thoughts and behaviors on a binary (i.e., behaviors as either adaptive or maladaptive, fears as either realistic or unrealistic) and instead described embracing the nuances of their clients’ lived experiences.

Relatedly, clinicians working in the specialty anxiety clinic emphasized the need to adapt exposure practices to explicitly consider their clients’ culture and context when developing hierarchies. Several clinicians discussed how aspects of clients’ identity-related stressors influenced their experiences of anxiety and, in turn, the difficulty of their fear hierarchy. Importantly, clinicians recognized the need to initiate conversations with their clients to determine, for example, how their experiences of racial discrimination may make certain exposures more difficult. Several researchers have shared case studies and clinical guidance that emphasize the importance of assessing relevant cultural and contextual factors and explicitly discussing them in relation to clients’ exposure hierarchies [31,69,70] to ensure that exposure is considered within clients’ lived experiences and values.

When applying Ex-CBT techniques within the context of clients’ lived experiences (e.g., poverty, immigration concerns, neighborhood safety, inadequate access to food and transportation), many clinicians reported the need to expand their therapeutic toolkits beyond traditional Ex-CBT techniques to include augmentations informed by holistic case conceptualizations of their clients. Clinicians discussed a host of augmentations, including connecting clients with case management resources to support their basic needs, validating their lived experiences (e.g., racism, discrimination) before attempting to restructure thoughts, and systems-informed strategies, such as advocating for the clients’ social, educational, and medical needs, and safety-planning prior to engaging in exposure. This holistic approach aligns with the emerging literature about the importance of culturally responsive care that attends not only to individual psychopathology, but also to the systemic factors that shape distress [31,37,69]. Importantly, recent effectiveness work suggests that incorporating these augmentations when needed does not negatively impact the success of Ex-CBT [15]. Overall, the findings from this study indicate a need for adaptations and augmentations that incorporate culture and context throughout all phases of anxiety and OCD treatment. Detailed clinical implications for each subtheme are reflected in Table 2 to further illustrate how community clinicians describe their approaches to providing person-centered culturally responsive care.

### 4.3. Limitations and Future Directions

This study provides preliminary evidence that clinicians are actively addressing the cultural and contextual needs of minoritized youth in anxiety and OCD treatment; however, some limitations are worth noting. While qualitative analysis yields rich data that capture clinician perspectives, it can also pose limitations, such as limited generalizability, and is more subjective in nature as themes are determined within the researchers’ perceptions of meaning and inevitably influenced by their individual lived experiences, worldviews, and programs of research. Additionally, we only present clinician-self report data, which may reflect clinicians’ intended behaviors rather than their actual practices; we did not include collateral data, such as observational data, or youth and caregiver reports of clinician strategy use. Additional measurement methods (e.g., observation, client and family reports) are needed. Moreover, the clinician sample represented mostly White, female clinicians. While this is consistent with the mental health workforce, it is necessary to understand the perspectives of diverse clinician populations. An additional limitation of this study is that clinician responses focused on retrospectively recounting instances of incorporating cultural and contextual factors. Future research should include tracking of practices inreal-time to more accurately measure use of strategies to address clients’ culture and context. Lastly, we did not examine the extent to which clinicians’ approaches predicted clinical outcomes; we are unable to conclude that the strategies reported are effective in clinical practice. Future work should examine the effectiveness of specific adaptations, augmentations, and process-based approaches, including the extent to which they predict client engagement and outcomes.

An important consideration for future research on person-centered culturally responsive Ex-CBT is the field’s prevailing emphasis on “flexibility within fidelity” [71,72] in the implementation of Ex-CBT. While the principle of permitting some level of adaptation while maintaining core components of EBT (e.g., prioritizing exposure for clients with anxiety and OCD) is well-intentioned, there also are critical limitations in our current evidence base resulting from lack of representation of youth with minoritized identities that must be acknowledged [1,10,73]. Specifically, there may be interventions that are not currently considered core components for certain youth simply because they have yet to be formally studied. For example, interventions highlighting cultural strengths and encouraging racial and ethnic socialization promote positive development and buffer negative mental health outcomes across racially minoritized groups [74,75,76,77]; however, these interventions have not yet been formally examined in the context of Ex-CBT. Clinicians working with minoritized clients are placed in a difficult position as they attempt to navigate adherence to fidelity while considering cultural augmentations that may fall outside the bounds of what is currently considered evidence-based. The absence of empirical validation for these augmentations should not be mistaken as an indication of ineffectiveness; rather, this reflects a gap in the research literature that urgently needs to be addressed through studies that center minoritized populations and examine person-centered culturally responsive clinical strategies. Future research should further explore how cultural and contextual factors interact with anxiety and OCD symptoms and use this information to build on approaches shared by clinicians in this study to develop and test culturally responsive anxiety and OCD treatment strategies for diverse client populations.

Additionally, future efforts should focus on clinician training that emphasizes person-centered culturally responsive strategies [78]. Many clinicians in this study highlighted specific strategies for incorporating culture and context but also reported receiving little to no formal training on how to conduct assessment of cultural factors nor how to adapt or augment EBT for culturally minoritized populations. Clinicians in this study largely described an ad hoc “learn as you go” approach and reported mixed confidence and consistency in implementing culturally responsive strategies. These findings align with the broader literature suggesting that clinician training on integrating culture and context remains underdeveloped and inconsistently implemented across graduate training programs and continuing education [78,79]. Moreover, there is a lack of rigorous research assessing the effectiveness of clinician training for delivering person-centered culturally responsive care and what specific components need to be included in training [78]. This study gives initial insight into the types of therapeutic process factors (i.e., cultural assessment, collaborative decision-making, discussing identity, pacing, trust) and specific adaptations and augmentations to Ex-CBT (i.e., nuance in determining realistic and unrealistic fears, considering cultural and contextual factors in hierarchy development, case management, advocacy) that may help clinicians incorporate person-centered culturally responsive strategies throughout treatment.

## 5. Conclusions

This study examines practice-based approaches to person-centered culturally responsive treatment for anxious and OCD youth. Clinicians working in a specialty anxiety clinic and clinicians working in general community mental health clinics described the need to understand their clients’ culture and context and noted ways in which they addressed these factors. Clinicians described incorporating culture and context through adapting existing treatment techniques, augmenting treatment with strategies not traditionally included in Ex-CBT, and utilizing process-based approaches, such as cultural assessment, collaborative decision-making, discussing identity, pacing, and trust. While the results from this study can help inform person-centered culturally responsive Ex-CBT practices, it only presents s clinician self-report data and does not analyze the effectiveness of clinicians’ approaches to incorporating culture and context. Thus, more research is needed to equip clinicians with the necessary skills to more fully meet their clients’ needs.

## Figures and Tables

**Table 1:** Therapist sociodemographic characteristics.

*n* = 16		
Age	M	SD
	32.2	5.9
Gender	n	%
Female	11	68.9
Male	5	31.3
Race/Ethnicity		
Black or African American	1	6.3
Asian	4	25.0
White	11	68.2

**Table 2:** Themes, subthemes, and clinical implications.

Themes	Subthemes	Clinical Implications
Therapeutic Phase: Culturally responsive assessment, case conceptualization, and treatment planning		
Developing a culturally informed conceptualization of the client and their anxious distress	Proactively and continuously assessing cultural context	Gather cultural information during initial sessions including information about stressors and strengths related to the client’s culture (e.g., their salient identities), and context (e.g., immigration, acculturation, poverty-related stressors, local community supports). ○ This can be supported by developed tools such as the Cultural Formulation Interview [44] or the RESPECT Toolkit assessment guidelines. Approach cultural assessment as dynamic and integral to the treatment process by continually broaching discussions of culture and context throughout treatment as it relates to their treatment progress and goals.
	Understanding family cultural norms and expectations and their intersection with anxiety and OCD	Assess for youth and caregiver values and differences in values within the family as it relates to their mental health and treatment. Maintain openness and curiosity about family roles and expectations by asking questions rather than making assumptions, especially if these differ from your own worldview. Determine the extent to which fears are normative within the family’s cultural norms and expectations by asking the client and their family what their familial norms expectations are.
Taking a holistic, systems-informed approach to treatment planning	Determining the appropriateness of Ex-CBT techniques within clients’ environmental context	Derived from your culturally responsive case conceptualization, determine the extent to which fears are proportionate to the client’s environmental context. This will help identify what to target with Ex-CBT and what to target with alternative strategies. ○ If fears are associated with maladaptive avoidance, or are disproportionate to the client’s culture or context, target with exposure. ○ If fears are primarily associated with adaptive avoidance (i.e., stem from cultural or contextual stressors such as those related to discrimination, immigration, food/housing instability, community violence), exposure may not be appropriate.
	Addressing environmental stressors and structural barriers	Prioritize ensuring basic needs are met (e.g., food, housing, physical and emotional safety) in treatment before progressing to Ex-CBT or other treatment techniques. Target environmental stressors with non-exposure techniques such as safety planning, problem solving, and case management. As needed, connect families with resources and culturally relevant community organizations (e.g., schools, healthcare providers, caseworkers, culturally relevant community organizations) to advocate for client needs and address environmental stressors.
Therapeutic Phase: Culturally responsive treatment delivery and process		
Aligning Ex-CBT techniques with client values and cultural traditions		Consider how Ex-CBT components (i.e., psychoeducation, cognitive restructuring, exposure) may center implicit assumptions (e.g., individualism, emotional expression, what constitutes adaptive functioning) and adapt content and delivery to better resonate with clients’ worldviews and communication preferences. ○ E.g., tailoring presentation of treatment rationales by either emphasizing research-based effectiveness or personal clinical experience depending on the family’s comfort and trust in research, ensuring selected exposure practices align with family goals and cultural norms. Incorporate family’s cultural strengths within the treatment plan. Involve religious leaders in the case of religious obsessions to align cognitive restructuring and exposure practice within cultural norms.
Incorporating social identity into exposure planning		Discuss with the client the ways in which identity-related stressors may influence their fears and how they influence steps on the hierarchy to guide planning of exposure practices. Be mindful of the resources (i.e., time, money) required to conduct out-of-session exposures; support families to build exposure practice and rewards into the family’s existing routine in a feasible way.
Engaging in collaborative decision-making and flexibility		Acknowledge the client and their family as the experts on their lived experience and ask for client and family feedback and perspective throughout treatment. Empower the client and their family to be active members of the treatment team by offering choice in treatment and continuously seeking their input.
Building trust	Building trust through self-disclosure	Use strategic, judicious self-disclosure of social identities, mental health experiences, and contextual stressors to build rapport and trust. Explicitly name shared or unshared life experiences and social identities to open the door for further conversation and trust building. Avoid oversharing, which can risk shifting the focus of treatment inappropriately away from the client.
	Building trust through recognition and validation of negative experiences	Acknowledge and validate client experiences of discrimination, marginalization, and mistrust in current sociopolitical contexts and in prior negative experiences with mental health care. Listen and validate the client’s experience without seeking to problem-solve or reframe, particularly when clients’ beliefs or fears are grounded in real, lived experiences of harm.
	Building trust takes time	Adjust the pacing of treatment to fit individual client needs (i.e., slowing the pace of treatment to prioritize rapport-building before introducing more directive therapeutic techniques, or maintaining the pace of treatment to earn trust by demonstrating the benefits of therapy).
Engaging in self-reflection as a tool to improve cultural responsiveness		Engage in self-reflection of how identities may impact desire to discuss culture and identity with clients or influence your interpretation of client and family emotional responses. Embrace cultural humility by being willing to engage imperfectly rather than avoiding conversations about culture and context. Incorporate discussion of cultural considerations into supervision to plan for each phase of treatment.

**Table 3:** Themes, subthemes, and additional illustrative quotes.

Themes	Subthemes	Quotes
Therapeutic Phase: Culturally responsive assessment, case conceptualization, and treatment planning		
Theme 1: Developing a culturally informed conceptualization of the client and their anxious distress	Proactively and continuously assessing cultural context	“That opens up the conversation about … what was it like for you growing up, what were your family’s values, how is that different from what’s happening now? So … making sure that they know we’re really interested and really care about their perspective and their culture and their experience.” (Specialty Clinic, Clinician 6) “A big part of [cultural assessment] is during the first initial meeting of really trying to get to know them. Or they just really giving them the opportunity to disclose [culture or traditions] if they feel comfortable. And then also … if they don’t feel comfortable disclosing with right away, leave a way to open for them to come back …” (Generalist Clinic, Clinician 8) “So, it really starts with the intake, because … we get a lot of identity, like you said, race, religion, sexual orientation, gender identity, all those things are kind of rolled into our … community mental health’s practice … and then in sessions … when I do information gathering, or just getting to know the person that it comes up.” (Generalist Clinic, Clinician 14) “I think my own progression has been from asking about it at the front end, and sort of thinking that the information I got at the front end was what I needed, to recognizing that this is actually really an ongoing process and continually assessing … [and] coming in with genuine curiosity and humility related to cultural factors.” (Specialty Clinic, Clinician 4)
	Understanding family cultural norms and expectations and their intersection with anxiety and OCD	“Conditions of worth [are] very important … growing up, what were expectations from family, society … the neighborhood you grew up in, the community… or your peers.” (Generalist Clinic, Clinician 10) “I had to really think about what my culture would say about differentiation from your family and what is appropriate … for her age.” (Specialty Clinic, Clinician 5) “If there’s religion-related OCD, compulsions, or fears or obsessions … I needed to understand enough to be able to say … is this an obsession and a compulsion that we need to address? … I talked to mom about it. I talked to [client] about it. We talked about the difference between her still being able to believe in God versus still being able to function in her household.” (Specialty Clinic, Clinician 1)
Theme 2: Taking a holistic, systems-informed approach to treatment planning	Determining the appropriateness of Ex-CBT techniques within clients’ environmental context	“I think an obvious example of this is a child who is fearful of police. And if they are Black, that fear is very different than if they are White. … If we just make assumptions of, oh, if every time you hear sirens, you have a panic attack, that must all be out of proportion, I think we’re doing a disservice to a lot of kids.” (Specialty Anxiety Clinic, Clinician 4) “She always felt like, on the train, that if somebody moved their bag, that they would have a weapon in there … that she would be a victim of violent crime at any time … and I didn’t want to push her and discount some of the real problems that women face in male violence, right? So, we … talked about why it might still be important [to take the train], what we’re going to keep an eye on … texting her parents if she feels uncomfortable, taking a phone call, carrying mace … things that were safety planning, rather than safety behaviors.” (Specialty Anxiety Clinic, Clinician 5) “I have a client right now where she and her mom believe in spirit guides, and she also talked about a fear of having a ghost in the house. And … disentangling … parts of that that might be an out of proportion fear. And … there may be things that are really legitimately scary that we wouldn’t target with exposure … I think about … what are the factors that might make somebody’s fears very different … than my assumption or my experience might be?” (Specialty Clinic, Clinician 4)
	Addressing environmental stressors and structural barriers	“I need to do my due diligence of making sure she does have access to food and care. So, it’s not … a situation where I would want to report her for neglect, because I knew Mom was working.” (Specialty Clinic, Clinician 1) “What’s been helpful is addressing the whole system, rather than just the individual … I spent a lot of time … coordinating care, so getting informants from school to give me data on what’s going on with youth, as well as any other pediatricians or religious leaders that could help me understand the nature of the impairment for the youth.” (Specialty Clinic, Clinician 7) “If there’s a caseworker on board, having them be part of it, or if the school is invested, having the school play a role in monitoring exposures or providing support. Definitely creating space for it in session too … And I think just in general, shoring up support resources, and not putting everything just on the caregiver, but thinking about the broader system that can support families.” (Specialty Clinic, Clinician 2) “I try to see [about] … the local English-speaking class for free, especially, or maybe they have some church … groups for people.” (Generalist Clinic, Clinician 11)
Therapeutic Phase: Culturally responsive treatment delivery and process		
Theme 3: Aligning Ex-CBT techniques with client values and cultural traditions		“With grief work, I found that taking that into account … what do they usually do, maybe it’s something that’s not really common in America … and then figuring out how we can bring some of that into their life … how to incorporate their traditions that they might have not been able to engage in.” (Generalist Clinic, Clinciain 14) “If I’m going to bring in a certain technique I do provide a lot of psychoeducation … and get a kind of feel of, like, okay, how can I truly adapt this? Is this area foreign, or it’s just not aligning with your culture …? So, just really being open to that as well … trying to … collaborate, to see how we can pick mini bits and pieces of this and … this other approach … it’s a trial and … we can always go back and try to see about another approach as well.” (Generalist Clinic, Clinician 8) “I was working with a Hispanic young woman, and in the cognitive restructuring portion, she talked about conflict with family and how it impacts her anxiety. And … they might have cultural expectations that would be considered enmeshed by some family therapists, and she was very anxious about feeling enmeshed for the rest of her life … So, in cognitive restructuring, we really had to … think about where we could be flexible but where it’s also important for her to meet some of her family’s expectations.” (Specialty Clinic, Clinician 5) “Being … thoughtful about what is the point of this intervention …? When I’m doing this intervention really well, does that align with the culture? … Cognitive restructuring challenge-based approaches may not always work as well with certain religions or cultures or family structures. And just being open and transparent, and if that doesn’t work, do we need to modify it or do we not need to use this thing because it doesn’t align with values?” (Specialty Clinic, Clinician 9)
Theme 4: Incorporating social identity into exposure planning		“I want to ignore what anxiety … is telling me, right? … But I don’t want to ignore cultural aspects of this person’s identity that might make an exposure hard or might make cognitive restructuring hard.” (Specialty Clinic, Clinician 5) “One kid [had] this fear of sticking out … at school. … I kind of pushed exposures on her in that direction with trying to … wear a dress you would wear for some kind of festive [event] … but have limited yourself from wearing because of this fear of sticking out … Her hair is not straight and blonde, and so she didn’t want to show it off … and we kind of did some things around that, which also kind of helped.” (Specialty Clinic, Clinician 1) “Recognizing that time … is one of the hardest resources to come by, especially for families of low SES. … So rather than having a contrived exposure, for example, like getting a sense of what their typical routine looks like, and how can we build exposure so that it doesn’t add burden to the family … and same with rewards … how can we make it work within the family resources, which includes time and parent energy? … And … our exposures often are requiring children to go into the community, but that may not be safe, depending on where the child lives.” (Specialty Clinic, Clinician 2)
Theme 5: Engaging in collaborative decision-making and flexibility		“I do believe clients are the experts of their own life. And really taking cues from them. So, when it comes to treatment goals, really asking them what is your goal? What would it look like for them? … Not just, like, oh, if you’re saying you want to do X, Y, and Z, this is my interpretation of how it should look like in your life. Maybe that’s going to look different.” (Generalist Clinic, Clinician 8) “I would … ask them, like, what do you actually want to work on? So, at least it gives them the autonomy to … decide … And then, usually … I’ll bring up the techniques I used to work with clients in the past. But I also will tell them, sometimes it works with people, sometimes it doesn’t … because everyone’s different … So, I would ask them, how do you feel if you try this? … So, that’s how we end up doing the treatment plan.” (General Community Mental Health Clinic, Clinician 10) “Approaching everything with an attitude of curiosity and not … assuming that a certain strategy or approach is going to work for a family or system, but really trying to … be really exploratory, both with respect to families’ resources and capabilities, and values and attitudes. And … continually modeling … [a] willingness to be collaborative and shift.” (Specialty Anxiety Clinic, Clinician 2)
Theme 6: Building trust	Building trust through self-disclosure	“Sometimes I use a little bit of self-disclosure about … [being an] immigrant … So, it’s helpful for them because it doesn’t feel like I see myself as a person with authoritarian role, but someone that’s equal with them … Sometimes I self-disclose about my experiences with anxiety, but I don’t go very detail about it … I’ll explain, like, I used to struggle with anxiety, and so, it helps them to know that … they’re not the only ones feeling that.” (Generalist Clinic, Clinician 10) “I was working with a Black family, and they had just transferred to me from another woman of color, I would make sure in the first session … to point out your previous therapist was a woman of color and I’m a White man. There’s definitely a difference in level of trust and level of privilege, and I just want to know how you guys feel about [it].” (Specialty Clinic, Clinician 5) “I acknowledge … my own identity as being a heterosexual White woman who doesn’t completely share their experience of the world …. I really try not to put the onus on them to teach me about it … I think there are some cases of very specific things where having a therapist who shares that experience, specifically, might be important. But … I’m willing to say, this is my experience, this is my lens, this is what I can offer. Hopefully, I’m the right person to help you with it. And if I’m not, I’ll find someone who is.” (Specialty Clinic, Clinician 4) “I have a client who is experiencing a very difficult family dynamic that I also went through for most of my life, and I wanted her to know she wasn’t alone in that … So, I shared that with her and … helped to validate her experience and provide her with some hope about her ability to overcome it … I think that when you can find common ground it’s a great opportunity to build rapport.” (Generalist Clinic, Clinician 16)
	Building trust through recognition and validation of negative experiences	“Being open and clear with them that I understand … [that] you learned to do these things, because your environment is unsafe, you’ve seen things … Once they feel heard … they start to be more open to working on it.” (Generalist Clinic, Clinician 14) “I was in a much better place to … collect information about … their values, or their racial identity … once they had established that I was in their corner … If I was willing to sort of work against some of the systems that were working against them and advocate for the family … it was then helpful for me to learn more about the family because that trust was there.” (Specialty Clinic, Clinician 2)
	Building trust takes time	“People … have had negative experiences with the consistency of the provider … parents not hearing from the provider … I found it creates a sense of mystery, distrust, not understanding what’s happening …. So, I found that consistency and transparency has been the best … motivator for building trust … It takes time for somebody to realize, like, okay, this isn’t changing. It’s a stable kind of professional relationship. It’s transparent.” (Generalist Clinic, Clinician 14) “Some kids have lived on the streets for the entirety of their lives and have no family members at their disposal … And I’m aware that, due to the nature of the trauma that our kids have experienced in their lives, sometimes a trusting relationship … may take a prolonged period of time, or sometimes it may happen instantly … or never.” (General Community Mental Health Clinic, Clinician 12) “There’s clients where rapport-building comes with just spending more time getting to know them. And then there’s other clients that are in real distress, and they’re not going to trust you until you show you can help them.” (Specialty Anxiety Clinic, Clinician 2)
Theme 7: Engaging in self-reflection as a tool to improve cultural responsiveness		“And once you enter the therapy room … examining your own bias … and then being thoughtful [that] … all aspects of your conceptualization and treatment are driven by the intersectionality of their various cultural identities. So, not pathologizing, but … using that as a way that understands and appreciate … their culture.” (Specialty Clinic, Clinician 9) “I want to be aware of the fact that there were differences between us … it’s always in the back of my mind … is there something that we’re disconnecting on? Is there a way that we can connect?” (Specialty Clinic, Clinician 1) “When I … have these conversations with clients, reflecting both before and after with my supervisor, how it went, and then depending on the client situation, I may even process in conversation with the client depending on their presenting problem.” (Specialty Clinic, Clinician 9) “And I think just also learning from missteps … being open about when you made a mistake in learning about a client and … being willing to be wrong and show that you’re a person too, that makes mistakes. And that I’m not above that or unapologetic but being open to … learning.” (Specialty Clinic, Clinician 2)

## Data Availability

The data presented in this study are available upon reasonable request from the corresponding author due to the sensitive nature of the qualitative data, which could contain identifying information although any protected health information has been removed.

## Abbreviations

The following abbreviations are used in this manuscript:

EBT	Evidence-based Treatment
Ex-CBT	Exposure-based cognitive behavioral therapy
OCD	Obsessive-compulsive disorder

## Appendix A

### Appendix A.1. Qualitative Interview Guide

Instructions: As a reminder if at any point you wish to skip a question or stop the interview, please let me know. Please refrain from using any identifying information about your clients including names or dates of service.

### Appendix A.2. Treatment Approach

1. Let’s start by imagining a child or teenager is (coming to you) seeking treatment for OCD or an anxiety disorder. What would your treatment approach look like?
  a If they mention deciding between modalities: Could you walk me through how you decide which treatment modality is best?
  b Probe: Could you give me examples of specific treatment techniques you typically might use?

2. What do you see as the biggest barriers to providing OCD and/or anxiety disorder treatment to youth at your clinic?
  a Probe: Barriers to treatment engagement (e.g., attendance, caregiver participation)
  b Probe: Barriers to treatment success (e.g., what do you see as getting in the way of youth with OCD/anxiety disorders responding to treatment?

3. How do you typically address these barriers to engagement or treatment success for youth with anxiety disorders or OCD in your clinic?

### Appendix A.3. Cultural Responsiveness

4. We are going to switch gears a bit to discuss cultural responsiveness. People define this term in many different ways. When you hear the term “culturally responsive therapy,” what does this mean to you?
5. In what ways, if any, do you assess for cultural or contextual factors (e.g., racial/ethnic identity, gender identity, socioeconomic status, beliefs about mental health and help seeking)?
6. Thinking specifically about working with youth with OCD or anxiety disorders, in what ways, if any, do you incorporate cultural or contextual factors into case conceptualization or treatment planning?
7. Have you ever had a situation in which a client expressed that therapy conflicts with their cultural identity or values? If so, please explain.
8. Sometimes our culture and background, such as the communities we belong to, the languages we speak, where we are from, our race or ethnic background, our gender or sexual orientation, our faith or religion, the neighborhood we grew up in, or how much money we have affect the way that we interact with people. Therapists and clients have aspects of their cultures and backgrounds that are similar and different. To what extent do you reflect on your own intersectional identity and how it influences your relationship with your patient and or the care you deliver?
  a Probe: How have similarities or differences in identity created challenges for you and your clients? Feel free to share any deidentified examples you have experienced that come to mind.
  b Probe: How have similarities or differences in identity benefited you and your clients? Feel free to share any deidentified examples you have experienced that come to mind.

9. In what ways have you focused on building relationship and trust with clients with different cultural backgrounds than your own. *can skip if needed*
10. How comfortable do you feel having conversations about a clients’ cultural background or identity, including experiences with racism and discrimination- and what has informed this comfort?
  a Probe *can skip if needed*: Has a client ever disclosed feeling misunderstood within the therapy context?

### Appendix A.4. Treatment Adaptations

If the clinician noted using Exposure-Based CBT in question 1, skip to question 14,

If the clinician did not mention using exposure and response prevention in question 1, ask:

11. Have you ever heard of Exposure-Based CBT? *If no go to #13*
12. Do you utilize exposure-based CBT with patients with anxiety disorders or OCD?
  a Probe: How do you use these strategies? Could you talk me through what it might look like for you to use exposure-based CBT with a client with OCD or an anxiety disorder?

13. If no, they have not heard of Exposure-Based CBT or are not sure, “Exposure-based CBT consists of intentionally helping youth face things they are afraid of and support them to reduce their use of avoidance or compulsive behaviors to ultimately help them learn to manage their distress more effectively. Exposure strategies can include having the client make a list of uncomfortable situations and rank those situations from easy to hard (i.e., build a hierarchy), helping the client gradually face his/her uncomfortable feelings (e.g., anxiety) in a supported way, and reduce the use of behaviors or compulsions that feel better in the short term but maintain anxiety in the long term.”
  a If they haven’t used it: What do you think about how useful or not this strategy would be for your patients with anxiety or OCD?
  b Probe: What, if anything would be missing?

14. If yes, they have heard of Exposure-Based CBT, but no they have not used it:Can you tell me little more about why you haven’t used Exposure-Based CBT and what treatment strategies you used instead?
15. If they have NOT used exposure-based CBT:You told me you use (List strategies they have reported using to treat youth with anxiety/OCD). How, if at all do you personalize these treatment strategies or processes based on your clients’ culture or background?
  a Probe: Treatment component they mentioned #1, 2, etc.

Only ask questions 16–18 if the clinician has used Exposure-Based CBT.

16. Okay, now I want you to think about whether and how you individualize/personalize treatment strategies to better fit your clients’ needs when delivering OCD or anxiety treatment. Standard general exposure-based CBT for anxiety disorders or OCD typically includes providing psychoeducation, hierarchy building, exposure practices, cognitive restructuring and relapse prevention. Please take a moment and think of some specific cases you treated in your clinic to help guide your responses. Of note, please do not share any identifying information with me about those clients. In what ways have you individualized/ personalized treatment strategies or used alternative treatment strategies to improve cultural and contextual fit for your clients (e.g., client/clinician cultural identity, neighborhood context, client values, religious beliefs, gender identity)? Specifically, how have you adapted / individualized:
  a Psychoeducation
  b Exposure
  c Cognitive restructuring
  d Other components of treatment
  e Probe: Did you find yourself adding any treatment strategies or material not typically in standard Exposure-Based CBT to address culture or context (e.g., strategies to address race, acculturation, discrimination)?
  f Probe: Did you find yourself removing elements of standard Exposure-Based CBT?

17. What components of Exposure-Based CBT do you find you adapt most often to fit your client’s cultural context, and why?
  a Are there any other ways you think of adapting treatment?

18. How do you make decisions about whether and how to individualize/ personalize treatment for your clients? *Summarize previous reasons for individualizing and ask for any additions*
  a Probe: Are there any specific symptoms youth may present with that might prompt you to consider incorporating cultural/contextual values more or less? If so, what?

Ask all participants

19. There are many cultural/contextual factors that affect treatment, like: beliefs about mental health and help-seeking; mistrust of providers; social identity and background; immigrant stressors; religion; racism and discrimination, so on. I’m going to send you a list of some of these examples in the chat. [Send this in the chat]: “Family and client beliefs about mental health and help seeking; Client’s mistrust of providers / health care system; Social identity and background (race, ethnicity, gender, SES, age, sexuality, language); Immigrant status/Acculturation stressors; Religion; Racism and discrimination; Social determinants of health (transportation, neighborhood safety, food/housing security, trauma exposure); Parents’ own mental health needs; Cultural values and beliefs.” Are there cultural or contextual factors that are missing from this list?
20. What are some of the most common factors that come up among your clients? What specific strategies do you use to address these factors or incorporate them into treatment? Please feel free to use de-identified case examples. (Factor #1 they mentioned, #2, etc.)
  a Probe if they’re having a hard time: For example, some clinicians might bring the parents or family into a session to create a treatment plan together that aligns with their values. Or when explaining the diagnosis or treatment, framing it in a way that fits with the client’s values. Another example might be consulting with religious leaders or traditional healers.

21. Have situations arisen where you’ve needed more support to address clients’ cultural or contextual factors in treatment for OCD or Anxiety disorders? If so, please explain. *can skip if needed*
22. What kind of specific training, supports, or additional strategies, if any, do you think are needed to help clinicians deliver culturally responsive treatment to youth with OCD and anxiety disorders?
  a Probe for specifics: What would this look like?

Sociopolitical context

23. People have different definition of antiracism, based on your own definition, what, if any, antiracism efforts are/were in place within your agency, for example policies and procedures, trainings, focus in supervision? *can skip if needed*

Additional Questions

24. We talked about a lot of things. If you had one key message that you would like me to remember from this conversation, what would that be?
25. What else would you like to share or any questions for me?
